# Editorial: Application for Nanotechnology for the Treatment of Brain Diseases and Disorders

**DOI:** 10.3389/fbioe.2021.743160

**Published:** 2021-08-30

**Authors:** Jia Li, Meng Zheng, Yan Zou, Pu Chun Ke, Miqin Zhang, William A. Banks, Bingyang Shi

**Affiliations:** ^1^ Centre for Motor Neuron Disease, Department of Biomedical Sciences, Faculty of Medicine and Health Sciences, Macquarie University, Sydney, NSW, Australia; ^2^ Henan-Macquarie Uni Joint Centre for Biomedical Innovation, School of Life Sciences, Henan University, Kaifeng, China; ^3^ Nanomedicine Innovation Division, The GBA National Institute for Nanotechnology Innovation, Guangzhou, China; ^4^ Australian Institute for Bioengineering and Nanotechnology, The University of Queensland, Brisbane, QLD, Australia; ^5^ Departments of Materials Science and Engineering, University of Washington, Seattle, WA, United States; ^6^ Division of Gerontology and Geriatric Medicine, University of Washington, Seattle, WA, United States; ^7^ Geriatric Research Education and Clinical Center, Veterans Affairs Puget Sound Health Care Center, Seattle, WA, United States

**Keywords:** nanotechnology, blood brain barrier, brain disease therapy, drug delivery, brain imaging

Most brain diseases are fatal with no effective therapeutic solutions currently available, contributing to major health issues globally. One of the major obstacles in overcoming the pathologies of these diseases is the existence of the blood-brain barrier (BBB), which physically separates the brain and the bloodstream (Profaci et al., 2020). The BBB blocks nearly all drugs from entering the brain and reaching the diseased cells and tissues at dosages sufficient to fulfil their therapeutic potential (Srikanth and Kessler, 2012).

In the past decades, various strategies including drug modification, novel delivery systems, transiently opening the BBB by physical or chemical methods and bypassing the BBB through intracranial or intranasal delivery (Li et al., 2021) have been actively explored to increase the brain uptake of therapeutics. Among them, nanoparticles-based delivery has emerged as a promising strategy owning to its simplicity of preparation, non-invasiveness, high efficiency and low toxicity (Li et al., 2021). Nevertheless, development in this area still lags far behind clinical requirements. Further improvement in the efficiency of nanoparticle BBB penetration faces considerable challenges–the fundamental mechanisms underlying the regulation of BBB integrity (Sweeney et al., 2016; Nation et al., 2019; Li et al., 2020) and the penetration of nanoparticles through the BBB are still unclear, hence smart nanoparticles that circumvent the selectivity of the BBB have yet to be developed.

This Frontiers Research Topic brings together contributions in new advancements in the mechanisms of the BBB regulation, the development of novel nanoparticles with the capability to be traced *in vitro* and *in vivo*, and the application of nanoparticles for treating various brain diseases.

Brain diseases are often associated with a dysfunctional often disrupted, BBB (Sweeney et al., 2018). Therefore, understanding the mechanisms of BBB regulation is critical to the development of novel therapeutics for treating brain diseases. In this special issue, Wang et al showed that microRNAs (miRNA) could either inhibit or enhance the expression of tight junction molecules, thereby directly regulating the BBB. In addition, miRNAs affected the structure and function of brain endothelial cells, including the cytoskeleton, channels and transporters of brain endothelial cells. Furthermore, miRNAs also targeted inflammation molecules and other molecules often used in the crosstalk between brain endothelial cells and the other cells of the neurovascular unit. The profound effect of miRNAs on BBB function and integrity makes them a promising target for treatment of brain diseases. Additionally, improvement of brain vascular function may benefit the treatment of brain diseases. In the article by Zhu et al, the authors developed amorphous selenium nanoparticles (A-SeQDs) for treating chronic isocarbophos poisoning through the protection of endothelial function. They showed that A-SeQDs inhibited inflammation while increasing oxygen saturation, leading to sodium hydrogen exchanger 1-dependent reduction of endothelial apoptosis. Consequently, isocarbophos-induced vascular dysfunction was inhibited.

The development of nanoparticles with the capability for imaging is critical to disease diagnosis and *in vivo* drug monitoring. Chung and Zhang developed a novel colloidal stable and non-toxic fluorescent probe, an iron oxide and carbon dot-based nanoparticle, to deliver chemotherapeutics for killing of cancer cells. This new type of nanoparticle was low in toxicity and able to respond rapidly for quantitative imaging. Transparent cranial implants provided a possibility for chronic brain imaging, thereby facilitating brain research. Halaney et al analyzed the optical properties of a ceramic, nanocrystalline Yttria-Stabilized Zirconia (nc-YSZ)-based transparent cranial implant. The optical properties of the implant were critical to the design of optical systems for imaging the brain and for interpreting imaging outcomes.

Nanoparticles have been employed to transport therapeutic agents to the brain, providing a safe and effective approach to improve brain drug delivery (Srikanth and Kessler, 2012). Ngowi et al summarized the unique properties of nanoparticles for diagnosis and treatment of brain diseases, including brain tumor, ischemic stroke, amnesia, and amyotrophic lateral sclerosis. The small size of nanoparticles, usually less than 100 nm, enables them to cross the BBB for delivering therapeutics and diagnostic probes to the brain parenchyma. In addition, nanoparticles can be modified for improved solubility, bioavailability and specificity of conventional drugs. To accelerate clinical translation, the authors highlighted the importance of determining the toxicity and bioaccumulation of nanoparticles in clinical settings. Khan et al discussed the application of various nanoparticles in treating Alzheimer’s disease (AD). They focused on the formulation of nanoparticles employed for this purpose, including organic, lipid-based, as well as metallic nanoparticles. The authors contended that the development of nanoparticles with multi-therapeutic capacities would be the research direction of future AD nanomedicine. Finally, in this special issue Li et al summarized the application of nanomedicine in AD and Parkinson’s disease by focusing on the pathogenic targets of nanoparticles, such as oxidative stress, protein fibrillation, and inflammation.
